# *Epichloë* Fungal Endophytes Have More Host-Dependent Effects on the Soil Microenvironment than on the Initial Litter Quality

**DOI:** 10.3390/jof8030237

**Published:** 2022-02-27

**Authors:** Zhen-Hui Yang, Ying Xing, Jian-Guo Ma, Yu-Man Li, Xiao-Qian Yang, Xiao-Bo Wang

**Affiliations:** 1 State Key Laboratory of Grassland Agro-Ecosystems, Center for Grassland Microbiome, College of Pastoral Agriculture Science and Technology, Lanzhou University, Lanzhou 730000, China; yangzhh19@lzu.edu.cn (Z.-H.Y.); xingy20@lzu.edu.cn (Y.X.); majg18@lzu.edu.cn (J.-G.M.); liym20@lzu.edu.cn (Y.-M.L.); yangxq21@lzu.edu.cn (X.-Q.Y.); 2 Erguna Forest-Steppe Ecotone Research Station, Institute of Applied Ecology, Chinese Academy of Sciences, Shenyang 110016, China

**Keywords:** plant–microbe symbioses, drunken horse grass, litter decomposition

## Abstract

Fungal endophytes have been extensively found in most terrestrial plants. This type of plant–microorganism symbiosis generates many benefits for plant growth by promoting nutrient availability, uptake, and resistance to environmental disease or stress. Recent studies have reported that fungal endophytes have a potential impact on plant litter decomposition, but the mechanisms behind its effect are not well understood. We proposed a hypothesis that the impacts of fungal endophytes on litter decomposition are not only due to a shift in the symbiont-induced litter quality but a shift in soil microenvironment. To test this hypothesis, we set-up a field trial by planting three locally dominant grass species (wild barley, drunken horse grass, and perennial ryegrass) with *Epichloë* endophyte-infected (E^+^) and -free (E^−^) status, respectively. The aboveground litter and bulk soil from each plant species were collected. The litter quality and the soil biotic and abiotic parameters were analyzed to identify their changes across E^+^ and E^−^ status and plant species. While *Epichloë* endophyte status mainly caused a significant shift in soil microenvironment, plant species had a dominant effect on litter quality. Available nitrogen (N) and phosphorus (P) as well as soil organic carbon and microbial biomass in most soils with planting E^+^ plants increased by 17.19%, 14.28%, 23.82%, and 11.54%, respectively, in comparison to soils with planting E^−^ plants. Our results confirm that fungal endophytes have more of an influence on the soil microenvironment than the aboveground litter quality, providing a partial explanation of the home-field advantage of litter decomposition.

## 1. Introduction

Plant–microbe symbioses exist widely in the grassland ecosystem. The symbiosis can exert great effects on both the growth and the physiology of host plants and on the microenvironment [1,2]. Most studies about microbial symbioses have focused on the mycorrhizal fungi and nitrogen-fixing bacteria due to their well-known beneficial effects on host plants [3,4,5,6]. However, the functional significance of other microbial symbioses, such as fungal endophytes, is much less understood to date [7,8]. Recent studies have shown that endophytic fungi play an essential role in enhancing the resistance and adaptability of host plants in grassland communities [9,10,11,12], but their potential impact on the host litter components and the soil environment across plant species has been largely overlooked [13].

Fungal endophytes are defined as plant-associated fungi that colonize, and live symbiotically within, plant tissues (e.g., leaves and stems) during a specific phase of their life. Generally, they are not harmful to their hosts when taking up residence in host organisms [8,14,15]. Fungal endophytes have been detected in approximately 30% of grass species [16]. They receive nutrients and protection from their host plants, and transmit from generation to generation by vertical transmission through host plant seeds [17]. In return, fungal endophytes protect their host plants from pathogens by producing secondary metabolites [18,19] and cell wall-degrading enzymes [20], or by inducing systemic resistance [21]. Moreover, they are capable of protecting their hosts against several environmental stresses [22] such as drought [23], salinity [24], nutrient depletion [25], flooding [26], and thermal stress [8]. As such, fungal endophytes increase their host’s fitness and they are likely to follow changes in their host’s morphological and physiological traits that are associated with nutrient acquisition, including a structural modification of plant tissues [27]. This may thus induce a shift in litter components or root exudates of host plants [28,29].

*Epichloë* is a typical genus affiliated with ascomycete fungi that commonly forms an endophytic symbiosis with grasses [30,31]. The symbiotic interaction between *Epichloë* endophytes and their hosts has been shown to affect many key ecosystem processes in different ways such as litter decomposition and soil nutrient cycling [32,33,34]. For example, *Epichloë* endophytes are able to induce a shift in chemical properties of aboveground host litter; and, consequently, they have an effect on litter decomposition [35,36]. The soil microenvironment tends to also be different between *Epichloë* endophyte-infected (E^+^) and -free (E^−^) plants due to host-induced root exudates, which thus strongly influence microbial decomposer communities by altering substrate quality and quantity [37]. Despite an increasing awareness of the fungal endophytes role in decomposition, few studies have been conducted to identify the mechanisms that fungal endophytes affect in litter decomposition [38].

In this study, we collected the aboveground litter and rhizosphere soils of *Lolium perenne* L. (perennial ryegrass), *Hordeum brevisubulatum* (Trin.) Link (wild barley), and *Achnatherum inebrians* (Hance) Keng (drunken horse grass), which have been demonstrated to form symbiosis with the *Epichloë* endophytes [39,40,41]. We hypothesized that foliar endophytic fungi would change the initial quality of the host litter and the soil microenvironment and that such an effect would vary across different host plant species. We aim to explore in the field (1) the shifts in litter quality and soil physicochemical and microbial properties across E^+^ and E^−^ status and plant species and (2) the differences in the effects of endophyte status and plant species on litter and soil properties.

## 2. Materials and Methods

### 2.1. Collection of Seed Material

The seeds of naturally occurring plants of wild barley (*H. brevisubulatum*) with mature reproductive tillers were collected from the Linze Experimental Station of Lanzhou University. The seeds of perennial ryegrass (*L. perenne*) Lanhei No. 1 were supplied by Lanzhou University. The seeds of drunken horse grass (*A. inebrians*) were harvested at maturity from symbiotic (*Epichloë gansuensis*, E^+^) and non-symbiotic (E^−^) plants grown in the experimental field of Lanzhou University. The selected wild barley, drunken horse grass, and perennial ryegrass with E^+^ status were infected by *Epichloë bromicola* [42], *Epichloë gansuensis* [43] and *Epichloë festuca var. Lolii* [41], respectively. The infection rate of individual plants was determined by microscopic examination of aniline blue-stained seeds. Plants with high (≥ 95%) and low (≤ 2%) colonization rates in the tillers were designated E^+^ and E^−^ seeds, respectively. Three plants were all screened for infection rates. These seeds were stored at a constant 4 °C in the lab before starting the experiment.

### 2.2. Field Experimenlt and Sampling

The field experiment was established in April 2017 and well maintained until December 2019 at the Yuzhong campus of Lanzhou University (Lanzhou, Gansu, 35°56′ N, 104°09′ E). The experimental site had a continental semi-arid climate and the mean annual precipitation and temperature were 400 mm and 6.7 °C, with an altitude of 1874 m. The soil type is classified as Huangmian soil [44,45]. Before the set-up of the field trial, the sod was removed and the soil was kept free of vegetation. The experimental plots (1 × 1 m) were arranged based on a split plot design. There were three blocks and within each block two replicated plots were randomly assigned for each treatment thus resulting in a total of 36 plots (3 plant species × 2 endophyte status × 6 replicates). The seeds of the wild barley, drunken horse grass, and perennial ryegrass with E^+^ and E^−^ status were planted in April 2017. The experimental field was regularly watered until the seedlings emerged. After two months growth of the seedlings, the two leaf sheaths of each plant for three species were collected and stained with aniline blue to observe the endophytic infection of the seedlings using the microscope [46]. We removed the seedlings that failed to be infected by *Epichloë* from E^+^ plots and replaced them with the successfully infected. The same method was applied to detect the E^−^ plot and ultimately ensure the infection rate of the seedlings in each plot reaching 100% (E^+^) and 0% (E^−^).

We collected the litter and the soil samples of three host plants from each plot in December 2019 (32-month growth and establishment period). Five plants were randomly selected from each treatment plot of each species, and an aboveground 5-cm segment was cut and collected as litter samples. The five plant litters collected in the same plot were put together as one composite litter sample per plot and placed in bags. Plant samples were taken to the laboratory, dried at 65 °C, and polished and homogenized before the chemical analysis. Five soil cores per plot (upper 5-cm layer) were collected and pooled to create one composite sample of each plot. Rocks, roots, and other debris were removed from the soil and immediately sieved (2 mm mesh size). The fresh sieved soil samples were then separated into three soil subsamples: one was for the measurement of soil moisture content; one was immediately stored in a 4 °C refrigerator for the analysis of microbial biomass carbon and nitrogen; and the remaining soil was naturally air dried for pH and chemical analyses.

### 2.3. Litter Quality Analysis

For litter samples, oven-dried litter mix samples from three grass species were ground into a powder with a ball mill (Retsch MM 400, Haan, Germany). The concentrations of total carbon (TC) and total nitrogen (TN) were determined using a Vario EL Cube (Elementar, Hanau, Germany) [47]. The content of total phosphorous (TP) was obtained colorimetrically by molybdenum antimony colorimetric methods after wet digestion in a mixture of HNO_3_, H_2_SO_4_, and HClO_4_ solution. The ratios of C:N, N:P, and C:P were then calculated based on these measurements. The initial levels of cell soluble contents, hemicellulose, acid detergent fiber (ADF), and acid detergent lignin (ADL) were obtained using an Ankom 2000i Fiber Analyzer (ANKOM Technology, Macedon, NJ, USA) [48].

### 2.4. Soil Property Analysis

For soil samples, 10 g of fresh soil was used to determine gravimetric soil moisture content by oven drying to a constant weight at 105 °C for 24 h. The soil pH was determined with a 1:5 soil-to-water ratio using a pH meter (PE-10, Sartorious, Germany). The soil mineral N was extracted using the solution 50 mL of 1 mol L^−1^ KCl solution with a 1:10 soil:water ratio and filtered through a filter paper. Using the indophenol blue spectrophotometric method and the UV spectrophotometry at 220 and 275 nm, respectively, NH_4_^+^–N (AN) and NO_3_^−^–N (NN) were then analyzed. Measurement at two wavelengths allowed for correction of interference by dissolved organic matter. The total soil carbon (TC) and the total soil nitrogen (TN) were determined using an elemental analyzer (Elementar Vario EL/Macro cube, Hanau, Germany). The total phosphorous (TP) was determined using the same method for litter samples. The available phosphorus (AP) was measured by molybdenum antimony blue colorimetry after acid digestion and the extraction of samples with 0.5 mol L^−1^ NaHCO_3_ (pH = 8.5) [49]. The soil organic carbon (SOC) was determined by the Walkley–Black wet digestion of a soil sample in a H_2_SO_4_-K_2_Cr_2_O_7_ solution. The soil microbial biomass carbon (MBC) and nitrogen (MBN) were measured using the fumigation–extraction method. The soils were extracted using the solution of 0.5 mol L^−1^ K_2_SO_4_ with a 1:4 soil: water ratio [50,51]. The MBC and the MBN were then calculated as the difference between unfumigated and fumigated subsamples with a proportionality coefficient of 0.45 for C, N [52]. All microbial biomass results were expressed on a dry weight basis.

### 2.5. Statistical Analysis

All data were tested for normality and homogeneity of variance in error before performing statistical analyses. The data was log transformed when necessary. Two-way analysis of variance (ANOVA) tests were used to identify the effects of endophyte fungi status, the litter quality, and the soil properties of plant species. One-way ANOVA and least significant difference (LSD) tests were used to check for significant differences in the litter quality and the soil properties between endophyte fungi status and among plant species. All statistical analyses were conducted on PASW Statistics 23.0. The bar graphs and the best of fit modeling of the regression between the soil nutrient and the microbial biomass carbon and nitrogen were produced using the Origin 2021 software (Origin Lab., Hampton, VA, USA). All data are shown as mean ± standard error of the mean and the differences were tested at the *p* ≤ 0.05 level. To exhibit the differences of the litter quality and the soil characteristics across plant species and endophyte fungi status, a multivariate data analysis was conducted using FactoMineR R package in R version 3.5.0 [53,54].

## 3. Results

### 3.1. Aboveground Litter Characterization

**Figure 1:**
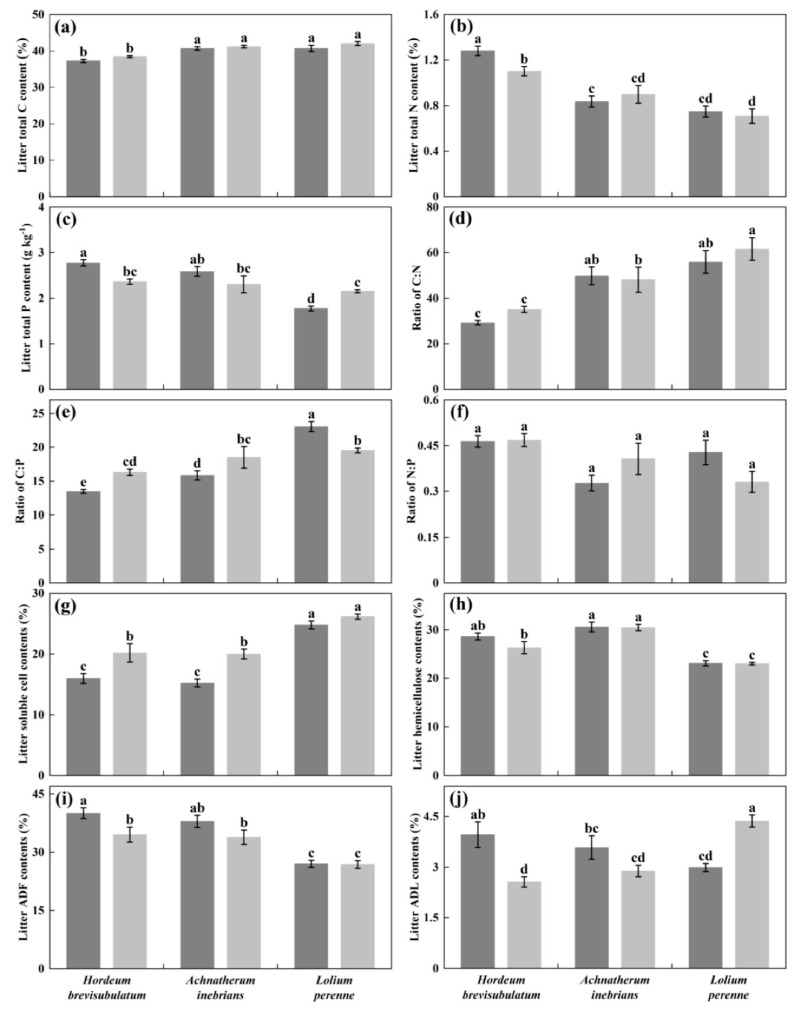
Effects of plant species (P) and endophyte status (E) on aboveground litter chemical components: (**a**) total C content, (**b**) total N content, (**c**) total P content, (**d**) ratio of C:N, (**e**) ratio of C:P, (**f**) ratio of N:P, (**g**) soluble cell contents, (**h**) hemicellulose contents, (**i**) acid detergent fiber (ADF) contents and (**j**) acid detergent lignin (ADL) contents of the wild barley (*Hordeum brevisubulatum* (Trin.) Link), drunken horse grass (*Achnatherum inebrians* (Hance) Keng), and perennial ryegrass (*Lolium perenne* L.) litter. Results are presented as mean ± SE (n = 6). Different lowercase letters indicate statistically significant differences (*p* ≤ 0.05) between *Epichloë*-infected (E^+^, black columns) and *Epichloë*-free (E^−^, gray columns) plant litter.

Plants species had a significant effect on nearly all measured initial litter properties including TC, TN, TP, C:N ratio, C:P ratio, N:P ratio, cell solubles, hemicellulose, and ADF (*p* ≤ 0.05) (Table 1). In contrast, endophyte status significantly affected TC (*p* = 0.027), cell solubles (*p* < 0.001), ADF (*p* = 0.003), and ADL (*p* = 0.001) (Table 1). The interaction between plant species and endophyte status was significant for TP (*p* = 0.001), C:P ratio (*p* < 0.001), N:P ratio (*p* = 0.047), and ADL (*p* < 0.001) (Table 1). The initial litter quality changed considerably among three grass species. The TC, C:N ratio, C:P ratio and cell solubles in wild barley were significantly lower than that in drunken horse grass and/or perennial ryegrass (*p* ≤ 0.05) (Figure 1a,d,e,g), while TN, TP, hemicelluloses, and ADF in wild barley were significantly higher than that in drunken horse grass and/or perennial ryegrass (*p* ≤ 0.05) (Figure 1b,c,h,i). The initial litter quality also differed between E^+^ and E^−^ status across each plant species. The cell solubles both in wild barley and drunken horse grass with E^+^ status significantly decreased compared with E^−^ status (*p* ≤ 0.05) (Figure 1g), while the ADF in both with E^+^ status significantly increased compared with E^−^ status (*p* ≤ 0.05) (Figure 1i). The ADL in perennial ryegrass with E^+^ status significantly decreased compared with E^−^ status (*p* ≤ 0.05), but it showed an inverse trend in wild barley (*p* ≤ 0.05) (Figure 1j).

### 3.2. Soil Chemical Properties

**Figure 2:**
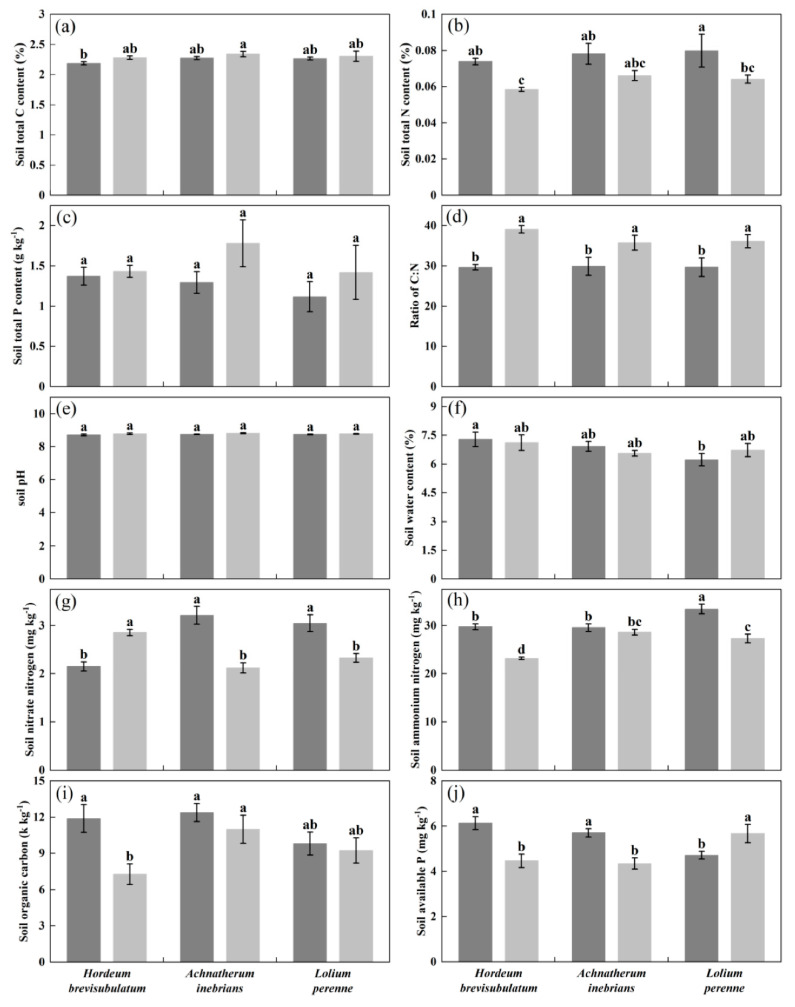
Effects of plant species (P) and endophyte status (E) on soil physicochemical parameters: (**a**) total C content, (**b**) total N content, (**c**) total P content, (**d**) ratio of C:N, (**e**) pH value, (**f**) moisture content, (**g**) nitrate-nitrogen (NN), (**h**) ammonium nitrogen (AN), (**i**) organic carbon (SOC) and (**j**) available phosphorus (AP) planting with the wild barley (*Hordeum brevisubulatum* (Trin.) Link), drunken horse grass (*Achnatherum inebrians* (Hance) Keng), and perennial ryegrass (*Lolium perenne* L.) soil. Results are presented as mean ± SE (n = 6). Different lowercase letters indicate statistically significant differences (*p* ≤ 0.05) between *Epichloë*-infected (E^+^, black columns) and *Epichloë*-free (E^−^, gray columns) soil.

Endophyte status significantly affected most of the measured soil chemical properties including TC (*p* = 0.001), C:N ratio (*p* < 0.001), SOC (*p* = 0.012), NN (*p* = 0.001), AN (*p* < 0.001), and AP (*p* = 0.005) (Table 2). By contrast, plant species only significantly affected AN (*p* < 0.001) (Table 2). The interaction between plant species and endophyte status was significant for NN (*p* < 0.001), AN (*p* = 0.001), and AP (*p* < 0.001) (Table 2). The soil chemical properties were distinctly different between E^+^ and E^−^ status across plant species. Soils with planting E^+^ plants had generally higher TN (*p* ≤ 0.05 for wild barley and perennial ryegrass), NN (*p* ≤ 0.05 for drunken horse grass and perennial ryegrass), AN (*p* ≤ 0.05 for wild barley and perennial ryegrass), SOC (*p* ≤ 0.05 for wild barley), and AP (*p* ≤ 0.05 for wild barley and drunken horse grass) content in comparison to the soils planting E^−^ plants (Figure 2b,g–j) but relatively lower TC and TP content, albeit not statistically significant (Figure 2a,c). The soil C:N ratio in E^+^ plant plots was significantly lower than that in E^−^ plant plots for each grass species (*p* ≤ 0.05) (Figure 2d).

### 3.3. Visualization of the Effect of Plant Species and Endophyte Status on Aboveground Litter and Soil Properties

**Figure 3:**
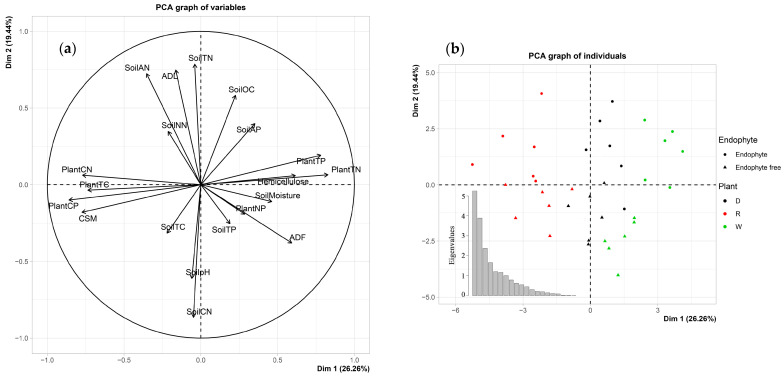
Variables (**a**) and individuals (**b**) graph in principal component analysis using PCA function in FactoMineR package. The first and second components explained 26.26 and 19.44% of the variance, respectively. TC: Total Carbon, TN: Total Nitrogen, TP: Total Phosphorus, CN: C/N ratios, NP: N/P ratios, CP: C/P ratios, AN: Ammonium Nitrogen, NN: Nitrate Nitrogen, SOC: Soil Organic Carbon, AP: Soil Available Phosphorus, CSM: Cell Soluble Materials, ADF: Acid Detergent Fiber, ADL: Acid Detergent Lignin. D: drunken horse grass (*Achnatherum inebrians* (Hance) Keng), R: perennial ryegrass (*Lolium perenne* L.), W: wild barley (*Hordeum brevisubulatum* (Trin.) Link).

The effects of plant species and endophyte status on aboveground litter and soil properties were more clearly visualized in Figure 3. The first and second principal components (PCs) explained 26.26% and 19.44% of the variance, respectively. The variables and the individuals map showed that plant species were distinctly separated along the first PC, and they affected mostly litter properties including TC, TN, TP, and cell soluble, etc., while endophyte status was distinctly separated along the second PC and affected soil-related properties including TN, AN, and C:N ratio, etc.

### 3.4. Soil Microbial Properties

**Figure 4:**
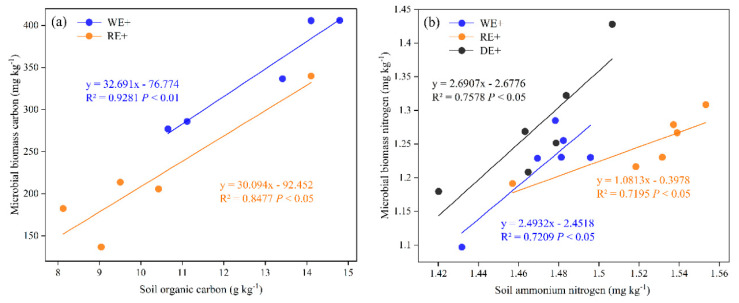
The linear relationship between (**a**) soil organic carbon (SOC) and (**b**) Ammonium Nitrogen (AN) and microbial biomass carbon (MBC) and nitrogen (MBN) in the *Epichloë*-infected (E^+^) soils across grass species.

Plant species and endophyte status significantly affected soil MBC (*p* = 0.007) and MBN (*p* < 0.001) (Table 2). The MBC in E^+^ soils was significantly higher compared with that in E^−^ soils (*p* ≤ 0.05). The MBC in soils with planting E^+^ wild barley and drunken horse grass enhanced by 16.28% and 10.42%, respectively, compared with that in E^−^ soils. The MBN (*p* < 0.001) varied similarly to MBC, being generally higher in E^+^ soils than in E^−^ soils. The MBN in soils with planting E^+^ wild barley and perennial ryegrass enhanced by 23.28 % and 25.88% compared with that in E^−^ soils, respectively. On average, MBC and MBN in E^+^ soil increased by 11.54% and 37.24% compared with that in E^−^ soil, respectively. Linear regression analyses were conducted to investigate the relationships between soil nutrients and the microbial biomass in soils with planting E^+^ plants across plant species (Figure 4). The MBC was correlated positively with SOC content in soils planting wild barley (*R*^2^ = 0.93, *p* < 0.01) and perennial ryegrass (*R*^2^ = 0.85, *p* ≤ 0.05), respectively (Figure 4a). The MBN was correlated positively with AN content in soils planting wild barley (*R*^2^ = 0.72, *p* ≤ 0.05), drunken horse grass (*R*^2^ = 0.76, *p* ≤ 0.05), and perennial ryegrass (*R*^2^ = 0.72, *p* ≤ 0.05), respectively (Figure 4b).

## 4. Discussion

The formation of plant–endophyte symbiosis generally reflects a mutualistic strategy to cope with environmental stress for symbionts. The plant–endophyte symbiotic interactions help to promote the coevolution of hosts and fungal endophytes [55,56], maintenance of biodiversity and plant and soil health [57,58]. In this study, we attempted to link the fungal endophyte status and its host-dependent effects to litter decomposition by endophyte-induced changes in litter and soil properties. We showed that the presence of the *Epichloë* in host plants increased the contents of soil available nutrients (SOC, AN, and NN). However, host specificity has a larger impact on litter quality than the effect of endophytic fungi. The findings provided insights into how the foliar *Epichloë* fungal endophyte symbiotic with wild barley, drunken horse grass, and perennial ryegrass affected the initial quality of litter in the host plant and the microenvironmental conditions of decomposition.

Most studies suggest that fungal endophyte–host plant interactions are mutualistic [59,60], but the interactions between the host–plant species and endophyte status are variable, ranging from positive to negative effects on litter decomposition (including litter quality and soil properties) [61,62]. The genetic factors of the plant species, endophyte status, and environmental factors can modify the nature of the symbiosis [63,64]. In this study, the effects of three host plants on litter quality and soil properties were inconsistent between E^+^ and E^−^ status. This is probably because the mutualistic symbioses depend not only on the presence of the endophyte but also on various abiotic factors and the network of species that interact with the host plant directly or indirectly [65,66]. The surveyed grass species and endophyte could thus play a decisive role in determining the nature of the grass–endophyte symbiosis.

We provided evidence for the effect of fungal endophyte on aboveground litter quality because of the significant differences observed in some litter chemical components between E^+^ and E^−^ status. A distinct increase in ADF and ADL content but a decrease in cell soluble content was generally found in our study. This finding is consistent with several previous reports showing that ADF or ADL increased within internal plant leaf tissues when plants are infected by fungal endophytes [67,68]. We cannot arbitrarily make a conclusion that plants infected by fungal endophytes may increase or decrease these chemical components because different plant species or species with different genotypes may respond completely differently to endophyte status. However, this endophyte-induced shift in host organisms may indeed indicate a response strategy of plant physiology in certain environmental conditions [69]. It is worth pointing out that aboveground litter properties are inclined to be mostly affected by plant species [70]. This is actually reasonable because compared with the endophyte-induced alternation of hosts organisms, the content of various chemical components in live and dead plant tissues are highly different among plant species [71].

Through this field experiment we surprisingly found that fungal endophytes had strong influences on most examined soil physicochemical parameters, particularly involved in soil nutrients such as SOC, AN, NN, and AP content, etc. This interesting finding provided an additional clue to link plants with different endophyte statuses to altered soil microenvironments. It is though difficult to identify direct or indirect relationships between them based on our current data set, such a correlation may suggest some potential processes. For instance, studies have shown that the quality and the quantity of root exudates of plants can experience great changes when they are infected by fungal endophytes [72,73], which can consequently lead to a shift in microbe-mediated soil nutrient pools. Alternatively, this linkage possibly resulted from interactions between the plant–soil microbiome for nutrient competence and transmissions [74]. Increasing the soil available carbon (C), nitrogen (N), and phosphorus (P) content in E^+^ plots across three plant species also suggests a beneficial effect of *Epichloë* endophytes on the host plants, in line with most previous reports [75]. In the long term, fungal endophytes may thus contribute greatly to plant and soil health in ecosystems. In contrast to endophytes status, plant species had very small and insignificant impacts on examined soil properties. This is not in accord with most studies conducted in grassland ecosystems [76,77]. The inconsistency may relate to similar physiological responses from selected plant species.

In general, litter decomposition is affected by two major factors including initial litter quality and the decomposition environment. Therefore, based on the findings we mentioned above, *Epichloë* endophytes may have a positive effect on litter decomposition processes via altering initial host litter and soil biotic and abiotic properties. Our data provided supportive evidence such as increased litter N and P contents and decreased soil C/N ratio, as well as significant positive correlations between increased soil nutrient and microbial biomass in E^+^ plots. Firstly, higher N and P concentrations have commonly indicated faster decomposition rates [78]. For example, previous studies have shown that the primary phase of litter decomposition was constantly positively correlated with the initial litter N or P concentration [79]. Secondly, litter N and P content, as primary energy resources for soil microorganisms, are often positively correlated with microbial activities in the decomposition process [80,81]. Hence, increased initial litter N and P concentration with E^+^ status probably suggest a beneficial effect on litter decomposition. Furthermore, the decreased C/N ratio and the increased microbial biomass resulting from increased nutrients in soils with planting E^+^ plants across three selected grass species provides further evidence to support this point as a number of studies have indicated their positive effect on promoting litter decomposition [82,83].

## 5. Conclusions

In conclusion, our findings verified the hypothesis that *Epichloë* endophytes did affect both the initial litter quality and the soil environment. Importantly, we showed that endophyte status had more host-dependent effects on soil biotic and abiotic factors compared with their effects on host litter properties. In contrast, plant species had only dominant effects on litter properties. The endophyte-induced shifts in soil nutrient availability and microbial activities could lead to a significant promotion of litter decomposition and thus assist our understanding about the home-field advantage of litter decomposition. Our findings suggest a new research direction in the future that could focus on performing studies involved in the impacts of key ecological processes and ecosystem functions induced by fungal endophytes.

## Figures and Tables

**Table 1:** Results of two-way ANOVA for the effects of plants species (P) and endophyte status (E) on initial litter quality. Statistically significant values at *p* ≤ 0.05 are shown in bold.

**Litter Quality**	**Plants Species (P)**		**Endophyte Status (E)**		(P) × (E)	
	***F*** **-Value**	***p*** **-Value**	***F*** **-Value**	***p*** **-Value**	***F*** **-Value**	***p*** **-Value**
TC	26.026	**0.000**	5.388	**0.027**	0.311	0.735
TN	38.131	**0.000**	1.369	0.251	2.463	0.102
TP	21.744	**0.000**	1.725	0.199	9.542	**0.001**
C:N	22.026	**0.000**	0.983	0.329	0.568	0.573
C:P	31.620	**0.000**	0.994	0.327	9.902	**0.000**
N:P	5.022	**0.013**	0.023	0.882	3.400	**0.047**
Cell solubles	50.594	**0.000**	23.335	**0.000**	2.180	0.131
Hemicellulose	43.715	**0.000**	1.686	0.204	1.254	0.300
ADF	27.503	**0.000**	10.436	**0.003**	2.429	0.105
ADL	2.777	0.078	13.252	**0.001**	12.316	**0.000**

**Table 2:** Results of two-way ANOVA for the effects of plants species (P) and endophyte status(E) on soil properties. Statistically significant values at *p* ≤ 0.05 are shown in bold.

**Soil Property**	**Plants Species (P)**		**Endophyte Status (E)**		(P) × (E)	
	***F*** **-Value**	***p*** **-Value**	***F*** **-Value**	***p*** **-Value**	***F*** **-Value**	***p*** **-Value**
TC	1	0.362	14	**0.001**	0	0.909
TN	1.391	0.264	3.294	0.080	0.197	0.822
TP	0.803	0.457	2.689	0.111	0.513	0.604
C:N	0.527	0.596	26.443	**0.000**	0.627	0.541
SOC	3.066	0.061	7.208	**0.012**	2.283	0.119
NN	1.253	0.300	12.539	**0.001**	28.063	**0.000**
AN	14.505	**0.000**	57.237	**0.000**	9.123	**0.001**
AP	0.510	0.605	9.400	**0.005**	13.597	**0.000**
pH	0.304	0.740	3.481	0.072	0.113	0.894
SWC	2.647	0.087	0.000	0.987	1.003	0.379
MBC	5.935	**0.007**	1.364	0.252	0.176	0.839
MBN	0.276	0.760	24.960	**0.000**	2.241	0.124
MBC:MBN	1.555	0.228	2.418	0.130	0.792	0.462

## Data Availability

The data presented in this study are available on request from the corresponding author.
